# Review of economic evaluations of mask and respirator use for protection against respiratory infection transmission

**DOI:** 10.1186/s12879-015-1167-6

**Published:** 2015-10-13

**Authors:** Shohini Mukerji, C. Raina MacIntyre, Anthony T. Newall

**Affiliations:** School of Public Health and Community Medicine, Faculty of Medicine, University of New South Wales, Sydney, NSW Australia

**Keywords:** Respirator, Facemask, Economic evaluation, Cost-effectiveness, Influenza, Tuberculosis

## Abstract

**Background:**

There has been increasing debate surrounding mask and respirator interventions to control respiratory infection transmission in both healthcare and community settings. As decision makers are considering the recommendations they should evaluate how to provide the most efficient protection strategies with minimum costs. The aim of this review is to identify and evaluate the existing economic evaluation literature in this area and to offer advice on how future evaluations on this topic should be conducted.

**Methods:**

We searched the Scopus database for all literature on economic evaluation of mask or respirator use to control respiratory infection transmission. Reference lists from the identified studies were also manually searched. Seven studies met our inclusion criteria from the initial 806 studies identified by the search strategy and our manual search.

**Results:**

Five studies considered interventions for seasonal and/or pandemic influenza, with one also considering SARS (Severe Acute Respiratory Syndrome). The other two studies focussed on tuberculosis transmission control interventions. The settings and methodologies of the studies varied greatly. No low-middle income settings were identified. Only one of the reviewed studies cited clinical evidence to inform their mask/respirator intervention effectiveness parameters. Mask and respirator interventions were generally reported by the study authors to be cost saving or cost-effective when compared to no intervention or other control measures, however the evaluations had important limitations.

**Conclusions:**

Given the large cost differential between masks and respirators, there is a need for more comprehensive economic evaluations to compare the relative costs and benefits of these interventions in situations and settings where alternative options are potentially applicable. There are at present insufficient well conducted cost-effectiveness studies to inform decision-makers on the value for money of alternative mask/respirator options.

## Background

Both the World Health Organisation (WHO) and the Centre for Disease Control (CDC) guidelines recommend the use of a mask in low-risk settings and a respirator in high-risk settings (e.g. during aerosol generating procedures) to protect healthcare workers (HCWs) from seasonal influenza [1, 2]. The use of a respirator at all times is also advised for HCWs caring for patients with suspected infectious tuberculosis [3, 4]. These measures are important to protect HCWs as well as to reduce the spread of respiratory infections within hospitals. This can help to reduce both the costs associated with HCW absenteeism and the costs of nosocomial infections.

Mask/respirator availability may also prove crucial in the context of newly emerging respiratory infections, particularly as some diseases such as SARS (Severe Acute Respiratory Syndrome) and MERS-CoV (Middle East Respiratory Syndrome Coronavirus) may initially have no vaccine or treatment available, leaving non-pharmaceutical measures as the only available protection for HCWs. For other diseases such as pandemic influenza, reliance on vaccines for protection is not always possible due to time delays in vaccine development, manufacturing and distribution [5]. Furthermore, the stockpiling of relatively expensive antivirals for influenza pandemics may not be cost-effective in low and middle income settings [6].

In the absence of standardised mask/respirator nomenclature [7], we will use the term ‘mask’ to indicate standard surgical masks, also referred to as “medical masks” in some countries. These are not specially engineered to protect the wearer from aerosol transmission of droplet nuclei and viral particles [8, 9]. ‘Respirator’ will be used to denote all personal protective facemasks engineered for filtration and fit to prevent the transmission of respiratory viruses and aerosol droplets. Several air purifying respirators that filter the inhaled air through filtering materials are available for use by HCWs [10], these include: N95, HEPA (high-efficiency particulate air), PARP (powered air purifying respirator), DM (dust-mist) and DMF (dust-mist-fume).

There are substantial differences in cost between different mask/respirator options. These cost differences may be an important determinant in the development of country specific mask/respirator guidelines for HCWs. For example, the more costly PARP is only recommended in the guidelines for high income countries [7]. Many countries’ guidelines recommend fit testing and training sessions for respirator use, but contain limited descriptions of what this should entail [7]. Fit testing can be qualitative or quantitative, with the latter being more expensive and adding a substantial cost to respirator use but not to mask use, which does not require fit testing [10].

Two studies evaluating mask/respirator use [11, 12] were included in a previous economic review of influenza pandemic measures [13]. There are a small but growing number of economic evaluations for masks/respirators. The aim of this review is to identify and evaluate this existing literature and offer advice on how future evaluations on this topic should be conducted.

## Methods

The Scopus database was searched for all English-language literature on the economic evaluation of mask or respirator use for the control of respiratory infection transmission. Scopus includes 100 % coverage of both MEDLINE and EMBASE [14]. The majority of the relevant literature identified in the initial Scopus search included influenza or tuberculosis as either the single focus or one of the diseases to control. For this reason, these terms (influenza OR tuberculosis) were used in *addition* to the generic terms related to infection and transmission, i.e. they did not prevent the identification of articles focused on other infectious respiratory diseases. The final search strategy contained publications until the 1st of August 2014, and used the search terms (as keyword, title or abstract): ‘facemask*’ (or ‘mask*’ or ‘respirator’ or ‘N95’ or ‘non$pharm*’ or ‘{personal protect*}’) AND ‘economic*’ (or ‘cost*’) AND ‘infect*’ (or ‘transmi*’ or ‘influenza’ or ‘tuberculosis’).

**Fig. 1:**
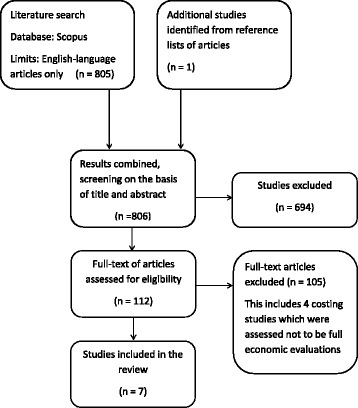
Flow diagram for study inclusion

The 806 results from this initial Scopus search were screened (Fig. 1). The abstracts of these articles were reviewed on the basis of whether they were economic evaluations (e.g. cost-effectiveness, cost-utility or cost-benefit studies), and whether the intervention being evaluated involved masks and/or respirators as a strategy to prevent the transmission of respiratory infections. From this process, 112 full text articles were considered potentially relevant and their full texts were further reviewed for eligibility. Of these, only six were confirmed to be economic evaluations of mask or respirator infection control interventions. One additional study by Dan et al. [12] was identified from the reference list of a systematic review [13]. These were the main studies used in our qualitative synthesis. Four other costing studies [15–18] have been published but these examined the cost of the intervention only and were not classified as full economic evaluations (i.e. they did not examine the health impacts or the cost savings from prevented illness). Data from the included studies was independently extracted intro a spread sheet by SM and this was reviewed by ATN. Disagreements were resolved by discussion between these authors.

## Results

### Description of economic evaluations identified

Seven economic evaluations of mask/respirator use in the control of respiratory infections were identified (Table 1) [11, 12, 19–23]. These studies varied widely in their settings and methodologies. The studies by Adal et al. [19] and Nettleman et al. [23] were published in 1994 and were on tuberculosis in healthcare settings. Five others (published post 2008) were focused on seasonal or pandemic influenza [11, 12, 20–22]. One of these by Dan et al. [12] also considered a SARS scenario. Other than this study, to date, no other mask/respirator economic evaluations have been identified that focus on SARS, MERS-CoV or other respiratory viruses (other than influenza). There were also no economic evaluation studies identified on mask or respirator use to prevent transmission of Ebola.

**Table 1:** Descriptive and methodological details of identified mask and respirator economic evaluations

First author, year, setting	Jones and Adida, 2013 [22], European contact rates	Chen and Liao, 2013 [21], Taiwan	Tracht et al, 2012 [11], USA	Dan et al, 2009 [12], Singapore	Cahill et al, 2008 [20], USA	Adal et al, 1994 [19], USA	Nettleman et al, 1994 [23], USA
Infection(s)	Influenza epidemic	Seasonal influenza	Influenza A(H1N1)pdm09	Influenza A(H1N1)pdm09, SARS, 1918 Spanish influenza	Influenza A(H1N1)pdm09	TB	TB
Mask(s) used	N95	Surgical mask	N95	N95	N95, surgical mask	Isolation mask, respirators: DM, HEPA with/without disposable filter	Surgical cup mask, respirators: DM, DMF, HEPA
Mask intervention	1 mask/person/day for duration of epidemic (90 days). Assumed respirator use begins when 0.05 % population infected	Surgical mask use and natural ventilation	N95 respirator use by a varied % of the population for the duration of pandemic, starting when 0.001 % symptomatic	Green 0: no intervention, Green 1: PPE for HCWs in contact with suspected cases, Yellow: full PPE for HCWs in high risk contact, Orange: PPE for HCWs in contact with medium risk	Monthly stockpiling and use for duration of pandemic	HCW program: respirators, fit testing and HCW medical evaluation	HCW program: 20 masks/8 h shift for HCWs visiting patients in isolation
Mask intervention effectiveness	Baseline effectiveness was 50 %. Intervention estimated to reduce probability of infection to 30 % or 70 % of baseline, depending on person-to-person contact rates	Not explicitly reported	Intervention estimated to be 50 % effective in decreasing susceptibility and 20 % effective for reducing infectivity	Exposure reductions of 50, 80 and 90 % with intervention. A 5 % failure despite use of protective equipment and isolation measures	Probability of transmission in 5 min encounter (varied for different % compliance for masks)	Not reported	Assumed respirator would prevent 25 % of HCW exposure to TB
Source of effectiveness data	Estimate derived from respirator assigned protective factor (APF = 10) [29]. This was adjusted for estimated lack of training, compliance and mask quality to give APF = 2 (i.e. mask 50 % effective)	Based on assumptions from previous study Chen et al. 2008 [33] where mask efficacies are assumed to be 60 %, 70 %, 80 %, or 95 % and are combined in the model with other control measures	Laboratory data, Lee et al. 2008 [52], and a randomised control trial by Aiello et al. 2010 [26] that found hand hygiene and facemask together were 35-51 % effective but not facemask use alone	No data cited for exposure reduction, these are assumptions. Failure rate estimate from a hospital simulation study Seet et al. 2009 [32]	Laboratory data from Balazy et al. 2006 [30] used to build particle transmission model	Reported none available	Reported none available
Type of economic evaluation	Cost-effectiveness analysis	Cost-effectiveness analysis	Cost-effectiveness analysis	Cost-effectiveness analysis	Cost-effectiveness analysis	Cost-effectiveness analysis	Cost-effectiveness analysis
Perspective	Policy developer view	Not stated	Not stated	Healthcare institution	Not stated	Not stated	Not stated
Primary outcome measure	Total costs of intervention	Unit cost per person, per year	Net savings compared to no intervention	Incremental increase in cost per death averted	Productivity loss to economy from absenteeism	Cost of respirator use per case prevented and per life saved	Minimum estimates of cost per life saved and cost per death averted
Intervention outcome measures	Cases	Cases	Cases, deaths, hospitalisations	Cases, deaths	Deaths, hospitalisation, outpatient visits, absenteeism	HCW PPD test conversion rates	Patients isolated for suspected TB, confirmed cases pulmonary TB in patients and active pulmonary TB in HCWs

*PPE* personal protective equipment, *PPD* positive protein derivative skin test, *TB* tuberculosis, *HCW* healthcare worker, *SARS* severe acute respiratory syndrome, *HEPA* high-efficiency particulate air, *PARP* powered air purifying respirator, *DM* dust-mist, *DMF* dust-mist-fume

The interventions evaluated by the studies included N95 respirators [12, 20, 22], HEPA, DM and DMF respirators [19, 23], and surgical masks [19–21, 23]. Amongst the studies that focused on influenza, two of the five, by Dan et al. [12] and Chen and Liao [21], integrated mask/respirator use in a scenario in conjunction with other non-pharmaceutical control measures e.g. natural ventilation [21] or isolation measures [12], as opposed to evaluating the use of masks/respirators exclusively. This makes it difficult to assess to what degree the mask/respirator aspect of the intervention is driving the cost-effectiveness, or if the results are more attributable to the other control measures included.

The settings of the evaluated studies varied more broadly for the influenza studies than for those on tuberculosis. Three of the five influenza studies were in a community setting [11, 20, 22]. Of the others, one considered control measures for both the community and hospital setting [12] and the last was set in a primary school [21]. All of the seven identified studies were set in the USA or other developed countries, limiting the generalisability of the findings, e.g. for decision making in low resource settings. The lack of studies in low resource settings has been cited as one of the major impediments to the use of cost-effectiveness analysis for pandemic influenza preparedness planning in this context [24].

### Intervention outcome measures

Four of the five influenza studies included the number of infection cases as one of the outcome measures of intervention effectiveness (Table 1) [11, 12, 21, 22]. The remaining influenza study quantified and reported the consequences of infection on other outcomes e.g. prevented deaths, hospitalisations, outpatient visits and absenteeism [20].

Both tuberculosis studies measured conversion rates of newly positive protein derivative tests in HCWs at study hospitals [19, 23]. A positive test indicates a 5–10 % chance that the individual will develop active tuberculosis in their lifetime [25]. One of the two studies also estimated the number of patients isolated for known tuberculosis per month, confirmed pulmonary tuberculosis cases in patients in the past year and active cases of pulmonary tuberculosis in HCWs in the last 5 years [23]. This additional information provided a more complete measure of the impact of the intervention.

### Cost-effectiveness outcomes

Only three [12, 19, 23] of the seven studies reported results as cost per unit of effect. The evaluation by Dan et al. [12] of influenza and SARS reported an incremental cost per death averted [12]. The other two were the tuberculosis studies, with one reporting the cost to prevent a single occupational case of tuberculosis in the next 41 years [19] and the other reporting the cost per tuberculosis case prevented and the cost per life saved [23]. Two of the pandemic influenza studies evaluating combinations of interventions in the community concluded that mask/respirator interventions were cost saving in a pandemic setting [11, 22]. The first estimated substantial net savings if 10 % of the population had worn N95 respirators during influenza A(H1N1)pdm09, assuming an effectiveness of 20 % [11]. The second estimated that surgical mask use at 60 % compliance would yield savings of $US100-250 million, far exceeding the estimated intervention cost of $US20 million [20]. The influenza modelling studies by Jones and Adida [22] focused on demonstrating the feasibility of the methods rather than reporting key economic results. The influenza study by Chen and Liao [21] calculated a cost per individual to implement control measures, but limited the inclusion of costs to intervention and outpatient treatments costs.

### Mask and respirator effectiveness or “strategy impact”

Approaches to incorporate intervention effectiveness amongst the seven studies varied (Table 1). Both of the tuberculosis evaluations by Adal et al. [19] and Nettleman et al. [23] reported that no efficacy studies were available and thus no specific measures of mask or respirator effectiveness were included in their evaluations. Nettleman et al. [23] assumed that in a best case scenario, HEPA respirators would prevent 25 % of HCW exposure to tuberculosis, but no data was cited to support this estimate. Adal et al. [19] implicitly assumed that all HCW cases of tuberculosis that may have been acquired from patients in isolation with active tuberculosis would be prevented by the intervention.

Of the five influenza studies, Tracht et al. [11] was the only study that attempted to utilise clinical efficacy data. They obtained this from a randomised trial in a university residence setting [26] that found a significant decrease (35–51 %) in influenza-like illness if hand hygiene and medical masks were used together compared to a control, but no difference for medical mask use alone. Tracht et al. [11] uses this result to estimate N95 respirator use provides a 50 % decrease in susceptibility to influenza A(H1N1)pdm09. This may not be robust as the clinical trial measured different outcomes (i.e. influenza-like illness, not influenza A(H1N1)pdm09 cases) and it assumes there is no difference between the efficacy of medical masks and N95 respirators based on the findings of Loeb et al. [27], although other trials have found evidence that there is a difference [28]. The study by Jones and Adida [22] derived efficacy estimates from the assigned protective factor (APF) measure for N95 respirators released by the National Institute for Occupational Safety and Health [29]. The APF is estimated from laboratory testing completed by this organisation, including laboratory testing of the quantitative measure of fit when the respirators are fit tested to ensure a protective seal around the wearer’s face. Cahill et al. [20] also used efficacy estimates from laboratory investigation data [30]. These estimates may have limited applicability to measuring real-world effectiveness [31]. Dan et al. [12] estimated baseline intervention effectiveness rates of 50, 80 and 90 % as protective equipment (including masks) and isolation precautions were increased. They did not cite data to support these estimates, however a 5 % failure rate where transmission still occurs despite the use of protective equipment and isolation measures, was obtained from a hospital simulation study [32]. Chen and Liao [21] applied a relative efficacy estimate of masks in combination with other control measures which was developed in a previous modelling study that did not cite supporting data for efficacy input assumptions [33]. Other than Tracht et al. [11], none of the analyses used intervention effectiveness estimates from clinical trials such as the mask/respirator randomised trials that have been published to date on interventions in community and healthcare settings [26–28, 34–38].

### Costs

Only two of the seven evaluations, by Dan et al. [12] and Jones and Adida et al. [22], explicitly stated the economic perspective from which they had conducted their evaluation, these being a policy developer view [22] and the healthcare institution perspective [12]. On the basis of costs included and excluded, four of the seven studies are most aligned with healthcare payer perspectives [12, 19, 21, 23] and the remaining three with a societal perspective [11, 20, 22] (e.g. they included some form of productivity costs). Both tuberculosis evaluations by Adal et al. [19] and Nettleman et al. [23], considered costs from a healthcare provider perspective, reflecting the objective of preventing the burden of nosocomial cases in HCWs and patients. The influenza studies [11, 20, 22] more frequently applied a societal perspective, consistent with the substantial economic impact of productivity losses due to influenza absenteeism [39].

The reporting of costs included in the evaluations was often not transparent. One evaluation by Dan et al. [12] identified the omission of the indirect costs of lost revenue from the cancellation of elective surgeries, decreased outpatient attendance, lost clinical teaching time and administration costs of senior staff meetings. These costs were not discussed in other influenza modelling studies where they may have been relevant [11, 20, 22]. The tuberculosis studies by Adal et al. [19] and Nettleman et al. [23] relied on hospital databases for the number of masks/respirators used, and multiplied this by unit costs to calculate total costs. Administration costs were most thoroughly reported in the tuberculosis study by Adal et al. [19], which included staff program planning time and staff medical evaluation time.

All of the studies that estimated a cost per case, included the cost of infected individuals [11, 12, 20–22]. For example, one influenza modelling study by Jones and Adida [22] assumed a fixed average cost for each infection. This may not always be an ideal method to cost resources used as the cost of an average case may vary amongst patients [40]. Tracht et al. [11] allocated a separate average hospital cost per day due to influenza A(H1N1)pdm09 infections for three age groups.

### Approaches to model adherence to interventions

Three of the studies evaluating the intervention in the community included the impact of compliance [11, 20, 22]. These included simple approaches, as used by Cahill et al. [20] of assessing 60 and 100 % adherence scenarios, as well as by Jones and Adida [22] where an estimate of 50 % compliance was assumed. Tracht et al. [11] conducted a more thorough sensitivity analysis by dividing the population into three age groups and testing three scenarios with different proportions of compliance in each group. No study attempted to use compliance rates from real-world investigations such as clinical trials [28, 36].

## Discussion

There were a relatively small number of economic evaluations of mask/respirator use that met the inclusion criteria and no existing studies were found that address the cost-effectiveness of these interventions in low-middle income countries. The seven evaluations identified have limited utility to advise decision makers on the value for money that masks and respirators offer compared with other health spending choices. This is due to the reporting of results in the form of intermediate outcomes (e.g. case prevented) and the analysis of combinations of masks/respirators with other protection measures in evaluations [12, 21, 22]. There was also limited inclusion of clinical data to inform the effectiveness estimates and the impact of intervention compliance in the identified studies.  Compliance has major implications for a mask/respirator intervention targeting HCWs [9]. For example, obtaining high adherence in HCWs has not been feasible in some high-income countries such as Australia [41]. Variation in HCW mask/respirator compliance has been observed between countries, such as the lower rates seen in UK hospitals compared to Hong Kong and Singapore during influenza A(H1N1)pdm09 [42]. Mask/respirator adherence is primarily driven by perceived susceptibility to the infectious disease threats present [43–48].

### What clinical evidence is there on comparative mask and respirator effectiveness?

Only one of the reviewed studies [11] cited clinical evidence to support their mask/respirator intervention effectiveness parameters. However, there is a growing body of research in this area. An initial systematic review on the topic reported pooled efficacy measures from case control studies for N95 respirators and medical masks of 91 and 68 % respectively for the prevention of respiratory viruses [49]. A weakness of this initial review was that the case control studies included in the meta-analysis focused exclusively on SARS [50]. As a consequence, it should not be assumed that the conclusions of this meta-analysis are necessarily true for influenza or other respiratory infections [51].

The updated edition of this review concluded that there was no evidence of a significant difference in effectiveness between N95 respirators and medical masks [50]. However, this conclusion was heavily influenced by a single cluster randomised trial by Loeb et al. [27] that compared N95 respirators and medical masks, both used selectively in high-risk situations. This trial contained only 446 HCWs and may have been underpowered to detect a difference between the arms [28]. Two larger trials not included in this review have been published [28, 36]. One of these by Macintyre et al. [28], involving 1669 HCWs, found significantly greater protective efficacy for continuous N95 use when compared to continuous medical mask use, but no evidence that selective N95 use was superior to continuous medical mask use for the prevention of clinical respiratory illness [28].

### Limitations

The limitations of the review include the restriction to English language articles used in the search of the Scopus database. Further potential limitations are that the identification and screening process was carried out predominantly by one of the authors (SM). Finally, the limited number of studies identified for inclusion made it difficult to make general conclusions about cost-effectiveness.

### Recommendations

One key recommendation is that future economic analyses should attempt to apply clinical mask/respirator efficacy data, preferably from clinical trials, where this data is deemed applicable to the infectious agent, intervention and setting, rather than using estimations derived from expert opinion or laboratory testing studies [31]. In some cases this may not be possible, for example, it is not appropriate to extrapolate clinical trial results for respiratory viruses to tuberculosis and for ethical reasons, randomised trial data cannot be collected on the effectiveness of a respirator intervention in HCWs for tuberculosis prevention. While the use of other types of efficacy data applied in the studies identified by this review have limitations, the results of the studies may still have validity. However, the underlying assumptions made in the analyses do need to be carefully considered.

Although economic evaluations have been conducted for the use of mask/respirator strategies in HCWs for tuberculosis, the way these evaluations should be used to inform policy needs to be carefully considered. Cost-effectiveness criteria should only be one part of the decision making process for any intervention and there may be specific cases where this factor should be given less weight. Economic evaluations would be of less relevance to a decision maker in situations and settings where a respirator would be used regardless of its cost-effectiveness, but they may be more useful in evaluating situations where these devices are not currently recommended in a given setting.

Future evaluations should report their results in such a way that would allow them to be easily compared to other non-pharmaceutical or pharmaceutical interventions (e.g. cost per quality-adjusted life year (QALY) gained). If an evaluation fails to report results in an appropriate format, this limits its use for decision making as it means that the value of mask/respirator interventions cannot be compared with spending on healthcare interventions targeted at other diseases [40]. However, in some cases the use of intermediate outcomes (e.g. cases prevented) can still be useful for comparing alternative ways of reducing specific infection events. This may provide useful information to decision makers.

Future analysts may also want to consider whether masks/respirators are cost-effective for use by HCWs in the seasonal influenza context, or if they are only cost-effective in extreme pandemic scenarios which may necessitate a stockpiled supply of masks/respirators.

## Conclusions

Although the WHO and the CDC recommend that HCWs use masks for low-risk influenza exposure and respirators for high-risk influenza or tuberculosis exposure [1–4], there is currently a lack of economic evidence to support these recommendations. Further robust economic evaluations on mask/respirator interventions are needed.

## Abbreviations

HCW: Healthcare worker
HEPA: High-efficiency particulate air
PARP: Powered air purifying respirator
DM: Dust-mist
DMF: Dust-mist-fume
